# The Correlation between Clinical and Demographic Parameters and Sickness Absence in Diabetic Employees

**DOI:** 10.3390/healthcare9101309

**Published:** 2021-09-30

**Authors:** Oren Zack, Irena Golob, Gabriel Chodick, Idan Perluk, Rachel Raanan, Shlomo Moshe

**Affiliations:** 1 Department of Environmental and Occupational Health, School of Public Health, Sackler Faculty of Medicine, Tel Aviv University, Tel Aviv 6997801, Israel; orenzack@gmail.com (O.Z.); hodik_g@mac.org.il (G.C.); rachelraananrr@gmail.com (R.R.); shlomo.moshe@gmail.com (S.M.); 2 The Department of Occupational Medicine, Central District, Maccabi Healthcare Services, Tel Aviv 7662029, Israel; golov_i@mac.org.il; 3 The Medical Division, Central Headquarter, Maccabi Healthcare Services, Tel Aviv 7662029, Israel; 4 The Department of Occupational Medicine, Jerusalem and Hashfela District, Maccabi Healthcare Services, Rishon Letzion 7662029, Israel; 5 The Public Health Services, Ministry of Health, Jerusalem 9101002, Israel

**Keywords:** sickness absence, insulin-treated diabetes, diabetes mellitus, fitness for work, employment, unemployment

## Abstract

*Objectives:* Diabetes mellitus is one of the most significant and prevalent chronic diseases. Individuals with diabetes can still encounter substantial difficulties in finding and keeping their job because of their condition. The purpose of this study was to examine the scope of diabetes-related absence from work and its relationship with variables such as type of employer, workload, the severity of illness, and type of treatment. *Materials and Methods:* We conducted a case-control study, including 220 diabetic patients and 230 controls. Information regarding absence from work was obtained by reviewing medical records, and general patient information was retrieved by conducting telephone interviews. *Results:* Patients with diabetes had, annually, more days of absence than non-diabetic patients (8.5 vs. 2.7, respectively p and lt; 0.001). Among diabetic patients, public-sector employees were absent more than private-sector employees (9.0 vs. 7.2 days, respectively, p and lt; 0.05). A positive correlation was found between workload (measured by stamina) and absence (Pearson correlation = 0.098, *p* = 0.04). Concerning the clinical variables, we found that employees suffering from diabetic complications exhibited higher absence rates (15.5 vs. 5.7 days, respectively, p and lt; 0.003). Parameters like HbA1c levels, patient age, disease duration, and type of treatment did not differ significantly amongst the groups with regards to absence rates. *Conclusions:* The main variables affecting absence from work were not medical but rather sociodemographic: education, workload, and type of employer. The results of this study reinforce the perception that well-controlled diabetic employees can be combined in most types of occupations without fear of increased absence from work.

## 1. Introduction

The worldwide prevalence of diabetes mellitus (DM) has risen dramatically over the past two decades, from an estimated 151 million cases in 2000 (representing 4.6% of the global population at the time) to 463 million in 2019 (representing 9.3% of the worldwide population). Based on current trends, estimates are that 700 million individuals will have diabetes by the year 2045 [1,2]. Individuals with diabetes can still encounter substantial difficulties, not necessarily evidence-based, to find and keep their job because of their condition [3,4,5]. Alongside potential misplaced prejudice by employers, several clinical factors that undermine capacity or safety may contribute to unemployment (complications or uncontrolled hypoglycemia) [6]. Even though DM treatment has significantly progressed in the past decade, few publications have dealt with the employability of diabetic patients. Previous studies have suggested that people with diabetes were more likely to be unemployed and have problems with their jobs than the general population [3,4,5,7]. However, only a limited number of studies have been conducted about absence from work in patients with diabetes. It is known that there are numerous variables affecting absenteeism, including age, income, education, and various psychosocial issues [8]. Most studies have focused on a comparison between a group of diabetic vs. non-diabetic employees and examined the extent of absence without reference to demographic or clinical variables affecting absenteeism [9,10,11,12,13,14]. Moreover, none of these studies reflect the significant changes seen over the past decade in the practice of clinical diabetology and the available drugs and insulin preparations in everyday use. These changes have affected diabetic patients and may have an impact on their employability.

Our study objective was to compare sickness absence between diabetic and non-diabetic employees and examine the extent of absenteeism from work concerning variables that were not examined till now, such as type of employer, workload, the severity of illness, and type of treatment.

## 2. Materials and Methods

This retrospective case-control study compared 220 diabetic patients and 230 healthy workers, both groups of working ages. The population from which the random sample was taken consisted of working diabetic patients treated at the Diabetes Institute of Maccabi Healthcare Services (MHS). The control group consisted of healthy patients randomly selected from a registry of patients brought in for health surveillance examinations to the Occupational Department of MHS. Controls were chosen from the registry mentioned above due to open access to their patient records. Controls were non-matched and consisted mainly of patients undergoing periodic health surveillance for ionizing radiation. Data regarding general sociodemographic parameters and working status were retrieved from the medical records provided by MHS for each employee. These data were further verified and completed (where applicable) using phone interviews. 

We analyzed personal and sociodemographic parameters like age, gender, education (defined as elementary school, high school, and university graduate), type of employer (public or private sector), and the vocational variable stamina, i.e., the ability to exert one’s self physically over long periods without getting winded or out of breath (taken from the Dictionary of Occupational Titles (DOT), US Department of Labor) [15]. Each vocation, including the control group, was ranked by the DOT database on a scale of 1–100. Since more physically demanding occupations were classified with higher stamina, we chose this variable as representing workload. Using standardized methods, we collected independent clinical variables of diabetic patients, including diabetic complications of any kind (macro-vascular including atherosclerosis with myocardial infarction, cerebrovascular accident, peripheral vascular disease, and micro-vascular including retinopathy, nephropathy, and neuropathy), disease duration (from date of diagnosis, years), diabetic control (estimated by the average of the last three consecutive measurements of HbA1c levels) and type of treatment (oral vs. insulin). 

We used the participants’ identification numbers to examine data regarding sickness absence certificates kept within their medical records in the MHS database. Sickness absence certificates were obtained from 1 January to 31 December 2008. The sickness certificate registry is a complete and unified registry, including all sickness absence certificates provided to the patient by any treating physician. The outcome was defined as the total number of sickness-absence days certified by all treating physicians during the follow-up period, i.e., 2008.

### Statistical Analysis

Data were analyzed using IBM SPSS statistics version 27.0. Continuous variables are presented as means (±standard deviation) and categorical variables as frequencies (in percentages). The univariate statistical analyses undertaken used Chi-squared tests for discrete variables, and *t*-test for continuous variables. We used the non-parametric Wilcoxon rank-sum test for comparing the number of days of absence from work (dependent variable). We used the Mann–Whitney test to compare the number of days of absence in light of the small sample size. Multiple regression analysis was used to examine the impact of potential confounding factors like age, diabetic complications, HbA1c values, and type of treatment, on sick leave of diabetic patients. We used BH corrections for multiple comparisons. All tests were applied two-tailed, and a significance level of 0.05 was chosen. 

## 3. Results

This study included a group of employees with diabetes (N = 220) and a control group of non-diabetic employees (N = 230). On univariate analysis, employees with diabetes were significantly more likely to be older (46.1 vs. 41.2, *p* < 0.001) and less likely to have an academic education (68% vs. 85%, *p* < 0.001) (Table 1). The study group was comprised of 184 type-2 (50.0% on insulin treatment) and 36 type-1 (94.4% on insulin treatment) diabetic patients. No significant differences were found for age by type of diabetes or diabetes treatment. 

Patients with diabetes had more days of absence than non-diabetic patients (8.5 vs. 2.7, respectively, *p* < 0.001). Differences were found throughout all age and education categories. Higher education was associated with lower sickness absence (2.5 vs. 7.1 days, *p* < 0.01). In addition, among diabetic patients, public-sector employees were absent more than private-sector employees (9.1 vs. 7.3 days, respectively, *p* < 0.05) (Table 2). In both groups, a positive correlation was found between workload (measured by stamina) and absence (Pearson correlation = 0.098, *p* = 0.04) (Table 3).

Diabetes complications were associated with a higher rate of work absence (15.7 vs. 5.7 days, *p* = 0.019), but no significant differences were found between HbA1c levels, disease duration, type of diabetes, or treatment type (oral vs. insulin) (Table 4).

Multivariant regression for sick leave of diabetic patients adjusted for age, HbA1c, type of treatment, and complications yielded significance only for the complications variable (β = 9.885, *p* = 0.006) (Table 5).

## 4. Discussion

I In this retrospective case-control study, we found that the days of absence amongst diabetic patients were 3.1 times higher than non-diabetic patients (8.5 vs. 2.7 days, respectively, *p* < 0.001). Among the clinical variables, only diabetic complications were found to be related to the rates of absence. The main variables affecting absence from work amongst diabetic employees were not different from known variables affecting absence amongst the general population. As seen from the demographic parameters, the study group of diabetic patients was older than the control group (46.1 vs. 41.2 *p* < 0.001) and less educated than the non-diabetic subjects (68% vs. 85% university graduates, *p* < 0.001). To overcome these differences, our analysis was based on group stratification, as presented in Table 2. 

Our finding of higher absence days amongst diabetics is consistent with several studies. Kivimaki et al. found that diabetic employees had a 2.15-fold excess risk of sickness absence compared with their colleagues without the chronic disease [4]. Skerjanc et al. also reported more days of absence in diabetic vs. non-diabetic workers (31.71 compared to 16.57 days per year, respectively) [5]. Poole et al. have shown a mean sickness absence of 32 vs. 20 days/year, respectively [12]. Dray-Spira et al. showed among 506/2530 (diabetic vs. non-diabetic patients) that sick leave difference increased from 16.4 days during the five years preceding diabetes onset to 28.5 days during the following 5-year period [13]. Nexø et al. observed significantly higher hazard ratios of sickness absence in type 1 diabetes (1.4) and type 2 diabetes (1.5) compared with people without diabetes [14]. In another study, diabetic employees lost a larger number of working days (13.3 vs. 5.7) [16]. De Backer et al. have shown an adjusted odds ratio for the association between diabetes and sick leave to be 1.52 for total sickness duration [17]. Norlund et al. found that the average number of sick days/year amongst diabetic patients was 21.4 vs. 9.4 in the general population [18]. 

As seen from our study results, the average number of sick days reported among the healthy population is relatively low compared to the studies reported above (2.7 vs. up to 20 days/year). We believe this is secondary to the strict management and limitations on sick days in Israel as compared to other developed countries.

We evaluated several sociodemographic parameters both in the study and control populations and analyzed the data according to varying age strata within each group. In the parameter of education, we found lower rates of absenteeism among workers with higher education vs. high-school education, albeit not statistically significant. The diabetic patients exhibited the same trend, with rates of absence among lower-educated patients being higher than higher-educated ones (11.8 vs. 7.1, respectively, NS) but with borderline significance. The above is consistent with several studies published over the years, showing that absences from work are inversely related to the number of years of schooling [11,19]. 

Concerning differences in absence rates by age amongst the two populations, our results indicate that diabetic patients have higher absence rates regardless of age group. Comparison of rates between subgroups of either study or control groups did not exhibit significant differences. Studies published on this issue have demonstrated inconsistent findings. We found one study that showed younger age to be correlated with more absenteeism [20]. Among the middle-aged, Lund et al. showed higher absence rates at ages 40–49 vs. 18–29 (RR = 1.68) [19]. The same group demonstrated lower absenteeism in the age group over 60.

On the other hand, several other publications have shown a positive correlation between age and absenteeism [21,22,23]. We found one study that demonstrated the same trend of higher absence rates with older age amongst diabetic patients; in this study, those younger than 30 years had only one-tenth of the sick days of individuals younger than 65 years of age [11]. The high rate of sick leave among younger populations is commonly perceived to be associated with dependent children [19,20]. Another explanation for lower absenteeism rates amongst the older-aged people is that employees in this age group who suffered from long-term or chronic illnesses will have already utilized one of the labor market exit options available for this age group; for example, disability pension, early retirement pension or old-age pension [19]. In the review by Dekkers-Sanchez [21], limited evidence was found for old age as a risk factor for absenteeism; however, no explanation was given. The authors emphasize, and we support their claim, that employment, social, and insurance conditions vary from country to country and may influence outcomes such as disability pension and work disability. 

We found that diabetic employees in the public sector were absent more than in the private sector (9.0 vs. 7.3 days, respectively, *p* = 0.043). This finding is consistent with several studies that exhibited higher absenteeism rates in the public vs. the private sector [20,22,24]. It should be noted that the public sector is characterized by higher workplace stability and a lower threat for job loss (i.e., dismissal) vs. the private sector. We estimate that these differences between the sectors lie in different work perceptions by employees of either sector.

We found a positive correlation between the absences from work and workload (measured by stamina) in both groups—the greater the workload, the higher the rate of absence (*p* = 0.04) (Table 3). Boedeker et al. examined a relationship between workload and absences in 42,000 employees [25]. According to their findings, the absences rate among the employees with high physical demands was 1.46 times higher than that of employees without physical demands. Lund et al. have examined absences among 5357 workers; long absence was defined as a period of sick leave that exceeded eight weeks. According to their findings, uncomfortable working positions, lifting or carrying loads, and pushing or pulling loads increased the risk of the onset of long-term sickness absence [26]. Similar findings were found in other studies [22].

We tested several clinical variables concerning absences amongst diabetic patients. There was no association between HbA1c levels and absences from work, with similar results for years of disease and treatment type. However, we have found that diabetic patients with complications were three times more likely to be absent (15.7 vs. 5.7 days, *p* = 0.019) than patients without complications. This finding should be judged with prudence as results were not age-standardized; nonetheless, the lack of significance regarding the somewhat correlated disease duration does lend credence to a possible tendency between complications and higher absence rates. In the study by Skerjanc et al., the number of sickness days among employees with long-term complications was 1.7 times higher (46.31 vs. 26.98) than that of employees without complications; results were not statistically significant [5]. In the study by Kivimaki et al., it was shown that, among employees with diabetes, having three or more non-cardiovascular comorbid chronic conditions was associated with a 2.68-fold increased risk of sickness absence [4]. Waclawski et al. had published a study on a small group of patients (*N* = 63) in 1991 (when diabetic control standards were less stringent) demonstrating a greater frequency of absence, a larger number of working days lost, and a higher average duration of absence among those diabetic workers with poor control compared to those with good control [27]. We could not infer from the paper whether the higher rates reported amongst the poorly controlled are reflective of concomitant complications and presume that this biochemical marker is an indicator of potential and not present complications. In their study on employees with diabetes, Ervasti et al. showed that low socioeconomic status, obesity, and job strain are linked to comorbidity and increased work disability in employees with diabetes [28], findings that correspond and relate to our results. 

This study, being records-based only, has several limitations. Controls were non-matched and chosen from a registry of periodic health check-ups due to restricted access to medical records. Another limitation is the lack of information regarding non-medical sick leave such as child sick leave, sick leave by declaration, and parent sick leave, which resulted from querying medical records only.

Socioeconomic variables were limited without access to marital status and income. 

The assessments made regarding employment were based on comparing work in the public vs. non-public sectors only; they did not address risk-prone occupations that could be affected by events of sudden incapacitation (i.e., hypoglycemia).

Since our study group is relatively small (*N* = 220), further research on a larger cohort is warranted to corroborate our findings and support our conclusions. 

Lastly, since our diabetic study group included a small percentage of insulin-treated patients, we advise to exercise prudence with the generalization of our study results; we believe that a follow-up study, with a higher rate of insulin-treated patients, designed prospectively and covering an extended period of follow-up (3–5 years), would help strengthen our conclusions. 

## 5. Conclusions

In summary, we found the main variables affecting absence from work amongst people with diabetes to be non-clinical, but rather sociodemographic or employment-associated factors such as education, workload (measured by stamina), and type of employer; all similar to the pertinent variables seen amongst the general population. Only one clinical variable (complications of diabetes) was directly related to the absences of diabetic patients, albeit, we lack more insulin-treated patients in our study, so the impact of the type of treatment was not examined thoroughly. 

The study results correlate with the current approach concerning the importance of careful balance and care of diabetic patients, even at the cost of a shift from oral to insulin therapy. This study confirms that, upon assessment of fitness for work for diabetic patients, diabetic complications are amongst the most valuable parameters to be taken into account. We believe that this study’s findings may impact health policy by encouraging diabetologists, occupational medicine experts, and primary care physicians to support DM patients in their effort to integrate into the labor market. Further studies with more insulin-treated patients and longer follow-up periods are needed to better understand the impact of different factors on diabetic patients’ employment.

## Figures and Tables

**Table 1:** Population sociodemographic characteristics.

Predictors	Diabetic Patients (*N* = 220)	Control Group (*N* = 230)	*p*
Age (years) average, range	46.1 ± 8.9 (range 24–69)	41.2 ± 9.1 (range 20–61)	<0.001
Age by type of diabetes and treatment			
Type 1 diabetes	37.97 ± 21 (N = 36)	--	
Insulin Tx	37.53 ± 8.08 (N = 34)	--	
Oral Tx	45.50 ± 9.19 (N = 2)	--	NS
Type 2 diabetes	47.7 ± 8.14 (N = 184)	--	
Insulin Tx	48.57 ± 8.28 (N = 92)	--	
Oral Tx	46.84 ± 7.94 (N = 92)	--	NS
Gender			
Male	94 (43%)	109 (47%)	NS
Female	126 (57%)	121 (53%)	
Education			
University graduate	150 (68%)	197 (85%)	<0.001
High school	64 (29%)	32 (14%)	NS
Less than high school	6 (3%)	1 (0.5%)	--

**Table 2:** Days of sickness absence along one year of follow-up by age, education, and employer’s type.

Predictors	Diabetic Patients (*N* = 220)	Control Group (*N* = 230)	*p* Value
Total (No. of days)	8.5 ± 20.2	2.7 ± 6.9	0.001
By age:			
<35 (N = 23 vs. 59)	9.5 ± 17.3	2.8 ± 7.8	0.01
35–44 (N = 62 vs. 89)	10.3 ± 23.5	2.2 ± 6.7	0.02
45–54 (N = 99 vs. 66)	7.1 ± 18.1	3.2 ± 7.1	0.1
>54 (N = 36 vs. 16)	8.2 ± 21.5	3.1 ± 7.1	0.35
Between groups			NS
By education:			
University graduate	7.1 ± 17.8 (N = 150)	2.5 ± 6.3 (N = 197)	0.01
High school	11.8 ± 25.2 (N = 64)	4.0 ± 9.3 (N = 32)	0.1
Elementary school	7.0 ± 11.6 (N = 6)	(N = 1)	
Between groups			0.877
By type of employer			
Public	9.05 ± 19.982	2.72 ± 6.966	0.001
Private	7.26 ± 20.813	Not available	

**Table 3:** Workload measured in stamina and correlation to days of sickness absence along 1 year of follow-up.

	Pearson Correlation	*p* Value
Diabetic patients (*N* = 220)	0.05	0.511
Non-diabetic patients (*N* = 230)	0.11	0.097
Both groups (*N* = 450)	0.1	0.04

**Table 4:** Clinical variables associated with absenteeism.

Clinical Variable	Diabetic Patients (*n*)	Days of Absence Per Year	*p*
Type of employer			
Public	150	9.05 ± 19.982	
Private	69	7.26 ± 20.813	0.545
Complications of diabetes			
Yes	61	15.69 ± 31.198	
No	157	5.73 ± 12.982	0.019
Disease duration (years)			
<5	83	8.6 ± 21.4	
5–9	51	8.4 ± 17.1	
>10	86	8.2 ± 21.3	0.821
Average levels of Hba1c			
<6% (<42 mmol/mol)	14	20.7 ± 39	
6–7% (42–53 mmol/mol)	43	6.2 ± 13.2	
7–8% (53–64 mmol/mol)	75	9.1 ± 22.2	
>8% (>64 mmol/mol)	88	16.3± 7.1	0.221
Type 1 diabetes	36	10.42 ± 21.156	
Insulin Tx	34	10.62 ± 21.702	
Oral Tx	2	7.00 ± 9.899	0.917
Type 2 diabetes	184	8.09 ± 20.011	
Insulin Tx	92	9.73 ± 24.213	
Oral Tx	92	6.45 ± 14.613	0.267
Type 1/Type 2	36/184		0.527
Type of treatment			
Oral treatment	94	6.46 ± 14.492	
Insulin treatment	126	9.97 ± 23.48	0.174

**Table 5:** Multivariant regression analysis for sick leave of diabetic patients adjusted for age, HbA1c, type of treatment and complications.

Variable	β	B (S.E)	*p*
Age (years)	−0.124	0.168	0.80
Type of Diabetes	0.962	4.253	0.82
Complications	9.885	3.087	0.006
Insulin Treatment	1.536	2.967	0.80

## Data Availability

The datasets generated and/or analyzed during the current study are not publicly available due to their clinical and personal nature. Study datasets are documented within patient medical records. Due to their personal nature, they are not readily available but are available from the corresponding author on reasonable request.

## Abbreviations

PHQ9	Patient Health Questionnaire 9
FFW	fitness for work
MDD	major depressive disorder
WA	working ability
OH	occupational health
OP	occupational physician
METs	metabolic equivalents
HPQ	Health and Performance Questionnaire
WPAI	Work Productivity and Activity Impairment
QIDS-SR	Quick Inventory of Depressive Symptomatology Self-Report
GHQ-12	12-item General Health Questionnaire
LTSA	Long-Term Sickness Absence
WLQ	Work Limitation Questionnaire
WMH-CDI	World Mental Health Composite International Diagnostic Interview
SPS-6	Stanford Presenteeism Scale no. 6
